# Salivary Duct Carcinoma: A Case Report With Cytological and Immunohistochemical Study and Review of Literature

**DOI:** 10.7759/cureus.80815

**Published:** 2025-03-19

**Authors:** Saloni Verma, Eram Khan, Fahad M Samadi, Madhu Kumar, Shalini Gupta

**Affiliations:** 1 Department of Oral Pathology, King George's Medical University, Lucknow, IND; 2 Department of Pathology, King George's Medical University, Lucknow, IND

**Keywords:** androgen receptor, immunostaining, metastasis, oncocytic-like, salivary duct carcinoma, salivary gland cancer

## Abstract

Salivary duct carcinoma is a rare, aggressive salivary gland neoplasm that is difficult to diagnose preoperatively. It shows limited clinical resolution of recurrent or metastatic cases after conventional conservative chemotherapy and/or radiation therapy. Here, we present the case of a 42-year-old man with high-grade carcinoma of the salivary gland with morphological and immunohistochemical characteristics consistent with salivary duct carcinoma, along with a succinct literature assessment.

## Introduction

In the 2022 WHO classification of salivary gland neoplasms, 21 malignant epithelial neoplasms are covered, one of which is salivary duct carcinoma (SDC). SDC is a rare malignancy comprising approximately 1-3% of all malignant salivary gland tumors. It is highly aggressive arising predominantly from major salivary glands, chiefly affecting older male patients and having a high death rate, local recurrence, and early distant metastasis. SDC was first described in 1968 by Kleinsasser et al. and was characterized as a distinct entity after three large series [1-4]. Radiographically, this high-grade malignancy typically appears with ill-defined borders, an irregular shape, and heterogeneous enhancement; calcifications may also be observed in some cases. It often exhibits invasion into adjacent tissues, areas of necrosis, and perineural spread, distinguishing it from other salivary gland tumors. Additionally, SDC has a high propensity for both nodal and distant metastases, commonly involving the lungs, bones, and liver [5].

Neoplasms like mammary analogue secretory carcinoma (MASC), oncocytic carcinoma (OC), epithelial-myoepithelial carcinoma (EMC), myoepithelial carcinoma (MC), adenoid cystic carcinoma (ACC), acinic cell carcinoma (AcCC), and metastatic squamous cell carcinoma (SCC) mimic SDC. Because of the striking histological resemblance and aggressive nature of MASC, it is crucial to differentiate SDC from MASC using an immunohistochemical study. Here, in this paper, we describe a case of SDC, emphasizing the morphological and immunohistochemical characteristics that were analyzed through fine-needle aspiration (FNA) and immunohistochemistry, revealing findings indicative of SDC, supported by a concise examination of existing literature.

## Case presentation

**Figure 1:**
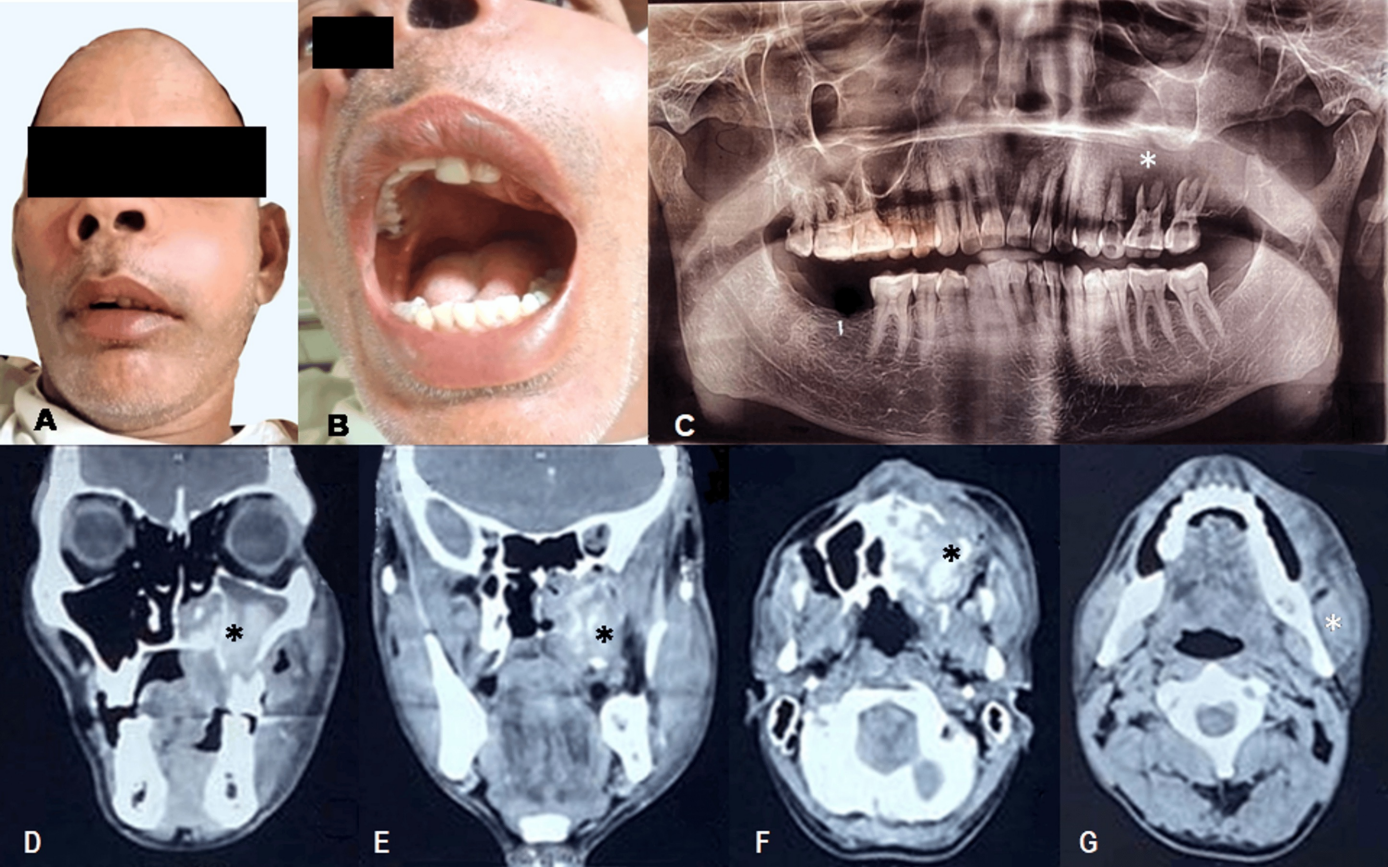
(A) Extraoral view. (B) Intraoral view. (C) OPG showing destruction of the floor of the left maxillary sinus (asterisk). D-G: CBCT of the head and neck findings. (D) CT image demonstrating an invasive, heterogeneously enhancing mass in the left parotid gland spreading into the sinus with the expansion of the left maxilla. (E) Ill-defined heterogeneous mass occupying the left maxillary sinus. (F) Poorly defined radiolucency with maxillary cortical expansion. (G) Tumor in relation to the left ramus and mandible (asterisk). OPG: orthopantomogram; CBCT: cone-beam computed tomography

A 42-year-old man presented with a rapidly enlarging swelling on the left side of his face measuring approximately 12.5×8.5 cm and extending from the left ala of the nose to 5 cm anterior from the left tragus anteroposteriorly and from the left medial region of eye infraorbital to 2 cm short from the left commissure superior-inferiorly, obliterating the left nasolabial fold with right side nasal deviation (Figure 1A). The swelling was normal in color, firm in consistency, non-transilluminant, non-fluctuant with diffuse borders, and fixed with tenderness with paralysis in the left infraorbital region. Submandibular lymph nodes were palpable bilaterally, firm, mobile, and tender on palpation; however, bilateral upper jugular lymph nodes were not tender. Intraorally, an erythematous diffuse swelling measuring 8×6 cm² covers a major part of the left hard palate, extending anteroposteriorly from the lingual vestibule of the anterior left maxillary teeth to the oropharynx and mediolaterally 2 cm right from the palate midline to the left maxillary buccal vestibule filling the right maxillary canine crossing the midline to the left maxillary second molar (Figure 1B). The swelling was firm, compressible, and tender; the left labial and buccal vestibules were tender; and there were grade I mobility in relation to 13-11 and grade III mobility in relation to 21-27. The left hard and soft palates presented with paresthesia. The CT scan demonstrated the presence of a lobular structured tumor in the left parotid gland, located adjacent to the left mandibular condyle with infiltrating, ill-defined margins (Figure 1C-1F). High serum alkaline phosphatase levels, a fall in serum sodium and ionic calcium, and elevated serum potassium were noted.

**Figure 2:**
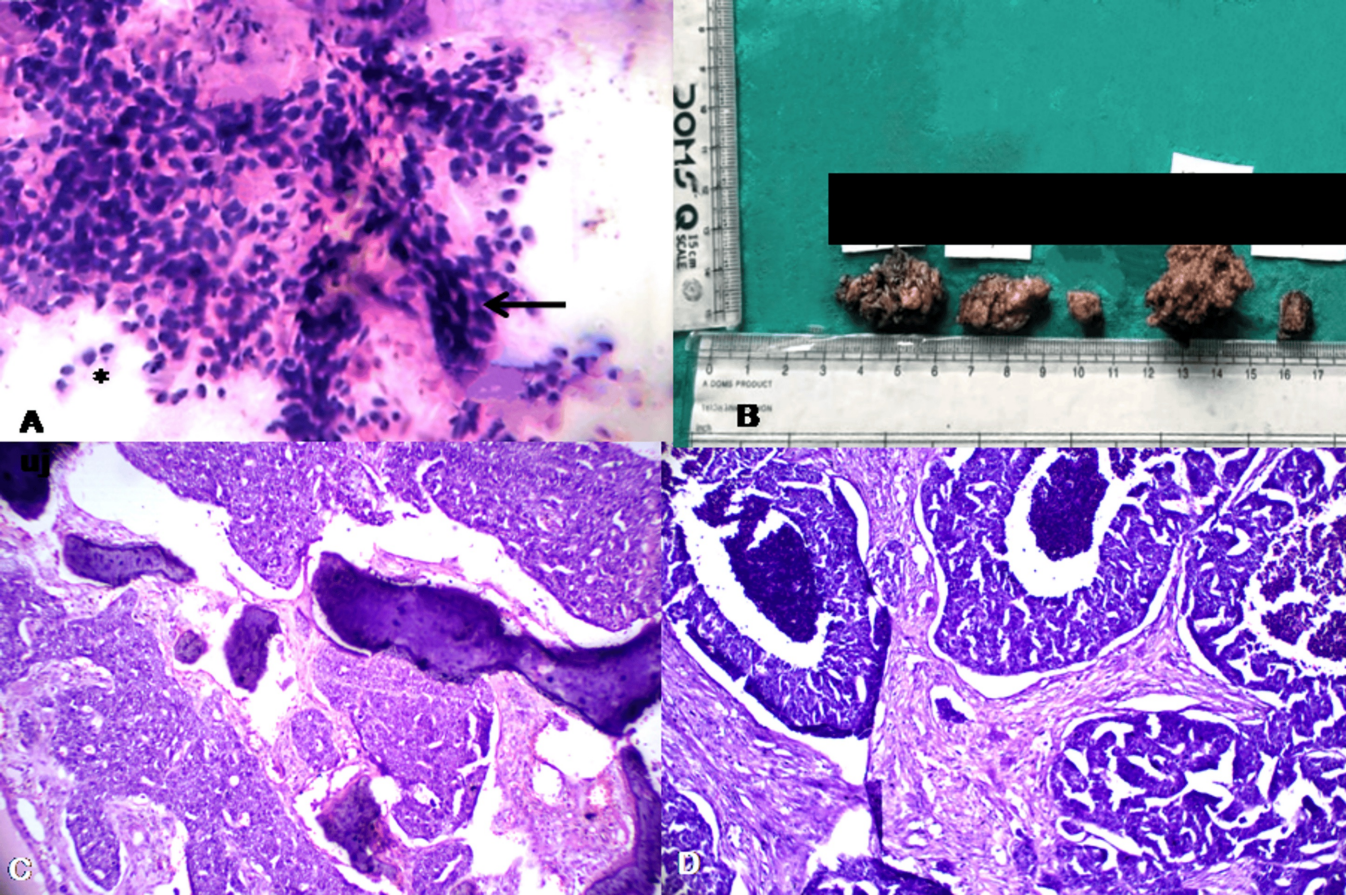
(A) Smear prepared from cystic fluid depicting hypercellularity with groups of large polyhedral epithelial cells (arrow) with pleomorphic nuclei showing discohesion (asterisk). (B) Resected specimen of the left maxillary region. C-D: Microphotographs of routine hematoxylin and eosin staining showing features of SDC. Tumor cells have prominent nucleoli with moderate to abundant eosinophilic cytoplasm. (C) Tumor cell proliferation in a cribriform growth pattern, ×100. (D) Intraductal component of SDC with comedonecrosis, ×200. SDC: salivary duct carcinoma

In fine-needle aspiration cytology (FNAC) smears, hypercellularity was noted, containing three-dimensional groups of large and polyhedral epithelial cells with nuclear pleomorphism and a high N:C ratio showing discohesion (Figure 2A). A diagnosis of high-grade carcinoma (Milan System) was rendered with a differential diagnosis of SDC and malignant oncocytoma.

The patient underwent two cycles of chemotherapy, following which radical left parotidectomy with regional lymph node dissection was done. The postoperative course was uneventful, and the specimen was sent for histopathological examination. The biopsy specimen contained three soft tissues measuring 3×2×1 cm, 3×1×1 cm, and 1×1×0.5 cm in size, brownish in color, and two hard tissue specimens (Figure 2B).

**Figure 3:**
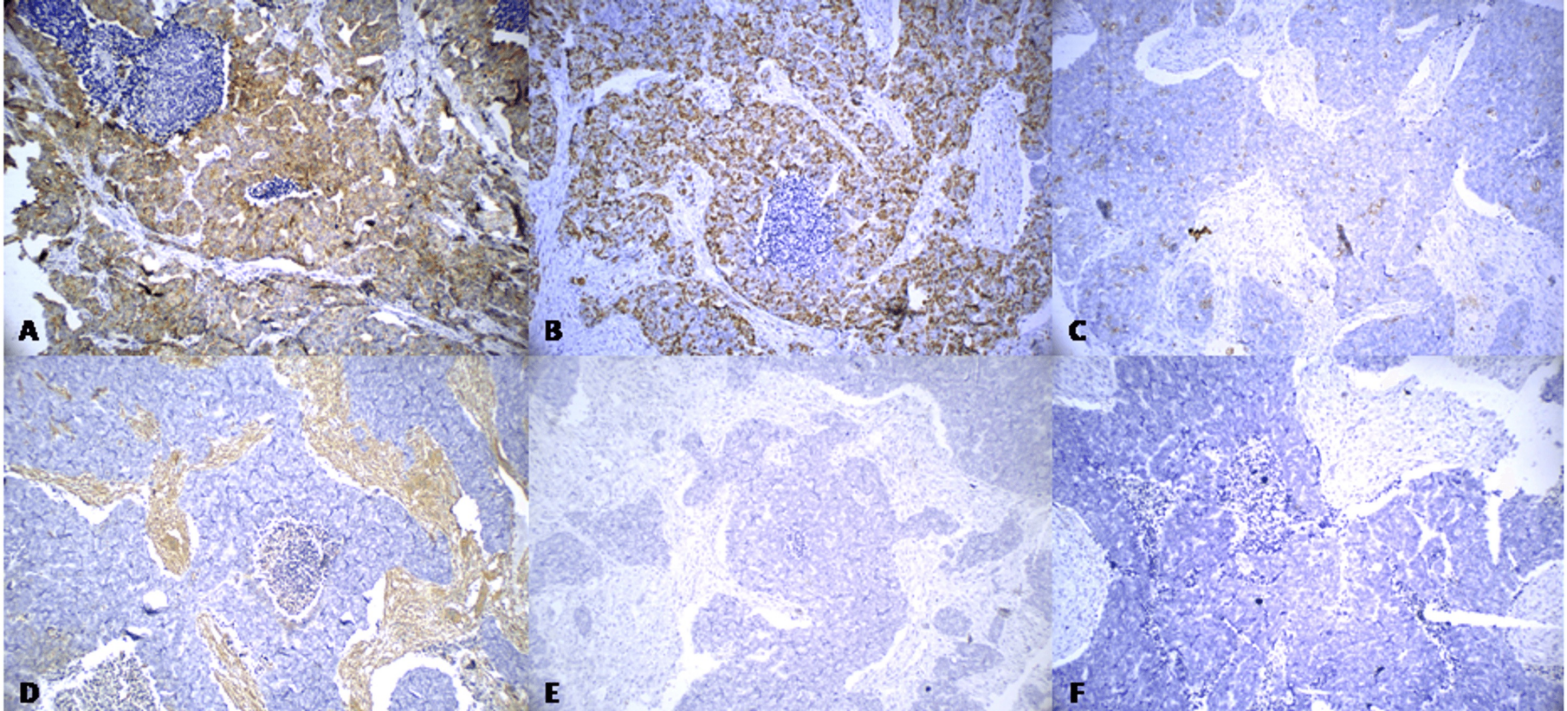
Immunohistochemical expression. (A) CK7+, ×200. (B) Ki-67+, ×200. (C) 34BE12 focal+, ×200. (D) Vimentin-, ×200. (E) S100-, ×200. (F) Synaptophysin-, ×200. CK7: cytokeratin 7; Ki-67: Kiel-67; 34BE12: high-molecular-weight cytokeratin (HMWK) antibody (cytokeratins 1, 5, 10, and 14)

Routine hematoxylin and eosin (H&E) staining demonstrated a combination of solid, cribriform, and trabecular patterns, exhibiting a prominent organoid growth pattern characterized by infiltrating cords, nests, and cribriform glandular structures within a desmoplastic stroma, along with focal areas of comedonecrosis. Individual tumor cells have large nuclei with pleomorphism consisting of conspicuous nucleoli surrounded by abundant granular eosinophilic cytoplasm (Figure 2C-2D). Immunohistochemistry had strong positivity for pan-cytokeratin (pan-CK), CK7, and Kiel-67 (Ki-67) (average Ki-67 labelling indices (LIs) ranging from 35% to 46%). High-molecular-weight cytokeratin (HMWK) antibody (cytokeratins 1, 5, 10, and 14) (34BE12) and epithelial membrane antigen (EMA) showed focal positivity (Figure 3A). Androgen receptors (AR) were positive, while estrogen receptor (ER), progesterone receptor (PR), P40, vimentin (Figure 3D), S100 (Figure 3E), human epidermal growth factor receptor 2 (HER2/neu) (which is not always negative), phosphotungstic acid hematoxylin (PTAH), and synaptophysin (Figure 3F) staining were negative. Therefore, we arrived at a final diagnosis of SDC. The patient could not complete subsequent chemotherapy and radiotherapy, which led to recurrence with metastasis, and the patient died one year after surgery.

## Discussion

SDCs are rare, very aggressive neoplasms affecting middle-aged and elderly people, with male predominance, frequently affecting the parotid, then submandibular, or other minor salivary glands [6]. Williams et al. in 2015 noted that about 25% of SDC arise from precedent pleomorphic adenoma as carcinoma ex pleomorphic adenoma and studied the role of viruses like Epstein-Barr virus, cytomegalovirus, and simian virus 40; ionizing radiation involvement; and increased hormone receptor expression in the development of salivary tissue tumors [7]. Symptoms can vary from massive swelling, pain with or without mastication, and speech problems in aggressive SDC cases to even asymptomatic slow-growing entities. During the typical progression of the illness and following the prescribed therapeutic regimen, complete or partial paralysis of the facial nerve is frequently encountered in as many as 60% of instances [1,5,6]. A diverse enhancement pattern, which is characterized by cribriform necrosis and infiltration into surrounding tissue, is a stronger indicator of SDC [7].

The metastatic spread has occurred in about 30% of patients at the time of presentation to the outpatient department and 50-60% of patients following diagnosis, and mortality is high. Metastatic disease can present as dyspnea, bone pain, or right upper quadrant (RUQ) abdominal pain due to lung, bone, and liver metastases, respectively [8].

Cytological features include large, round to oval islands of tumor cells with microcystic spaces, a cribriform pattern with monomorphic polygonal cells, pleomorphic and atypical mitotic figures, spherical nuclei, background necrosis, and copious amounts of finely granular cytoplasm, leading to the following differential diagnosis: adenocarcinoma not otherwise specified (NOS) and mucoepidermoid carcinoma (high grade). The individual cells show a marked degree of cytologic atypia suggestive of high-grade salivary gland malignancy according to the histological risk stratification model of SDC adapted from Nakaguro et al.'s Novel Histologic Risk Stratification Model risk groups (see Appendices) and show strong similarity to breast invasive ductal carcinoma [9].

Ki-67, HER2, PR, and ER are routinely checked to diagnose SDC where HER2 comes positive in 25-50% of cases and PR and ER are negative. Masubuchi et al. and Simpson et al. proposed an additional set of AR, CK, tumor protein 53 (p53), and epithelial growth factor receptor (EGFR) as AR positivity is highly characteristic [10,11]. Our case was CK7, AR (apocrine phenotype) positive. HER2/neu is not always negative and is usually positive in 25-50% of SDC cases. Cases lacking HER2 amplification have other molecular alterations, such as AR positivity or tumor protein p53 gene (TP53) mutations. SDC shows characteristically high AR expression (78-96%) in primary and metastatic tumors (94% and 93%, respectively) [12]. Previous literature showed 78-96% AR immunopositivity and supports androgen deprivation therapy with goserelin [12]. Fushimi et al. conducted a phase II trial on combined androgen blockade with leuprorelin and bicalutamide in patients with recurrent or metastatic salivary gland cancer, especially SDC, and they discovered that this regime has equivalent efficacy and less toxicity for AR-positive recurrent/metastatic or unresectable locally advanced cases compared to conventional chemotherapy, with an overall response rate and disease control rate of 42% and 86%, respectively, and a median overall survival of 30.5 months [13].

OC differs from SDC as it demonstrates abundant cytoplasmic granules, which are mitochondria, which are PTAH and anti-mitochondrial antigen stain positive or can be appreciated via electron microscopy [14]. EMC can be distinguished from SDC by the presence of a dual-cell population in well-differentiated components or throughout the neoplasm, wherein myoepithelial cells are highlighted by tumor protein 63 (p63) and smooth muscle actin (SMA) [14]. MC can be distinguished from SDC by plasmacytoid cells and specks of hyalinized stroma with strong and diffuse p63 and S100 expression [14]. ACC shows a distinct cribriform pattern with basement membrane-like material-filled pseudolumina and is negative for HER2, ER, and PR and shows a characteristic myeloblastosis proto-oncogene-nuclear factor I B (MYB-NFIB) fusion gene [14]. AcCC shows zymogen granules, which are periodic acid-Schiff (diastase), and is discovered on gastrointestinal stromal tumors 1 (DOG1) positive while being negative for HER2, ER, and PR [14]. Metastatic SCC (especially non-keratinizing) can be reliably distinguished from SDC with AR and p63, as nearly all SDCs are AR positive, while all SCCs are p63 positive [15]. SDC can be distinguished from other head and neck tumors by the fact that SDC fulfills the CK7+/CK20-phenotype, whereas SCC shows the CK7+/CK20+ phenotype [15,16].

Adenosquamous carcinoma shows glandular differentiation and a hint of keratinization with strong and diffuse p63 expression, while large cell undifferentiated carcinoma (LCUD) lacks a glandular pattern and is gross cystic disease fluid protein 15 (GCDFP-15) and AR negative. TP53, retinoblastoma 1 (RB1), suppressor of mothers against decapentaplegic 4 (SMAD4), Harvey rat sarcoma viral oncogene homolog (HRAS), adenomatous polyposis coli (APC), phosphatidylinositol-4,5-bisphosphate 3-kinase catalytic subunit alpha (PIK3CA), and G protein subunit alpha Q (GNAQ) were among the genes with recurrent somatic alterations in SDC, and the myeloblastosis proto-oncogene-NHS-like 1 (MYB-NHSL1) fusion gene was also noted, which can aid in further differentiating SDC from adenosquamous carcinoma and LCUD [17,18].

Fan et al. hypothesized that because SDC showed positive prostate-specific antigen and prostate acid phosphatase in 58% and 17% of their patients, respectively, it resembles characteristics of prostate cancer. These characteristics could bolster the idea that individuals with metastatic SDC may benefit from anti-androgen medication [19].

MASC shows micro-follicular growth with lower-grade areas having colloid-like secretions and no necrosis. Histopathological and molecular profiling should be carried out to give efficient diagnosis and therapy because of the morphologic and molecular similarities with breast cancer. The presence of ETS variant transcription factor 6 (ETV6) translocation by fluorescence in situ hybridization (FISH) differentiates MASC from SDC. Validation will be performed using Ki-67, HER2, PR, and ER, with AR, CK, p53, and EGFR utilized as supplementary markers [10].

Radiation treatment is used after an extensive surgery that involves the dissection of suspicious lymph nodes. Chemotherapy is only used for SDC metastatic stages. Approximately one-third of patients experience local recurrence, and almost 50% experience distant metastases despite rigorous surgery and subsequent radiation treatment. Usually, within five years, 65% of SDC patients pass away [20].

Recent research findings indicate that the overexpression of HER2 is linked to a poor prognosis. Genetic events have been seen in SDC like loss of heterozygosity linked to the expression of the EGFR family (e.g., HER2), and p53 can be employed for targeted immunotherapy [8]. Consequently, targeted treatment should be taken into consideration in the adjuvant situation, and HER2 status should be assessed at least in the presence of advanced SDC.

## Conclusions

We report a case of SDC affecting the parotid gland showing morphologic predominant solid with striking organoid pattern and CK, AR, GCDFP-15, and C-erbB-2 positivity, typical of SDC. While FNA biopsy is a good and accurate method for diagnosing SDC as a malignant neoplasm, it is not as good at differentiating it from other types of tumors. It is an aggressive entity with high mortality that mimics many salivary gland neoplasms and should be differentiated to aid in early diagnosis, make the right therapeutic decision for effective treatment, and improve the patient's survival.

## Appendices

**Table 1:** Salivary duct carcinoma: histological risk stratification model H&E: hematoxylin and eosin Table Source: Adapted from the Novel Histologic Risk Stratification Model of Nakaguro et al. [9]

Presence of the following factors	Total number of positive factors
Factors present showing adverse prognosis	1. Prominent nuclear pleomorphism
	2. ≥30 mitoses/10 high-power fields
	3. Vascular invasion (H&E stain)
	4. ≥5 poorly differentiated clusters
­Risk groups depending on the total number of positive factors	0, 1: low risk
	2, 3: intermediate risk
	4: high risk
